# Human Renal Fibroblasts, but Not Renal Epithelial Cells, Induce IL-1β Release during a Uropathogenic *Escherichia coli* Infection In Vitro

**DOI:** 10.3390/cells10123522

**Published:** 2021-12-13

**Authors:** Ashok Kumar Kumawat, Geena Varghese Paramel, Kartheyaene Jayaprakash Demirel, Isak Demirel

**Affiliations:** 1 Cardiovascular Research Center, Faculty of Medicine and Health, Örebro University, 701 82 Örebro, Sweden; ashok.kumawat@oru.se (A.K.K.); geena.paramel@oru.se (G.V.P.); 2 School of Medical Sciences, Örebro University, 701 82 Örebro, Sweden; 3 Public Dental Service, Region Örebro County, 703 64 Örebro, Sweden; kartheyaene.jayaprakash-jayaprakash@regionorebrolan.se; 4 iRiSC—Inflammatory Response and Infection Susceptibility Centre, Faculty of Medicine and Health, Örebro University, 701 82 Örebro, Sweden

**Keywords:** UPEC, human renal fibroblasts, inflammasome, UTI, IL-1β

## Abstract

Understanding how uropathogenic *Escherichia coli* (UPEC) modulates the immune response in the kidney is essential to prevent UPEC from reaching the bloodstream and causing urosepsis. The purpose of this study was to elucidate if renal fibroblasts can release IL-1β during a UPEC infection and to investigate the mechanism behind the IL-1β release. We found that the UPEC strain CFT073 induced an increased IL-1β and LDH release from renal fibroblasts, but not from renal epithelial cells. The UPEC-induced IL-1β release was found to be NLRP3, caspase-1, caspase-4, ERK 1/2, cathepsin B and serine protease dependent in renal fibroblasts. We also found that the UPEC virulence factor α-hemolysin was necessary for IL-1β release. Conditioned medium from caspase-1, caspase-4 and NLRP3-deficient renal fibroblasts mediated an increased reactive oxygen species production from neutrophils, but reduced UPEC phagocytosis. Taken together, our study demonstrates that renal fibroblasts, but not renal epithelial cells, release IL-1β during a UPEC infection. This suggest that renal fibroblasts are vital immunoreactive cells and not only structural cells that produce and regulate the extracellular matrix.

## 1. Introduction

Urinary tract infection (UTI) is one of the most common infections in humans and it is primarily caused by uropathogenic *Escherichia coli* (UPEC) [1,2]. The majority of the UTI are uncomplicated local infections. However, the infection can develop into a complicated UTI that can lead to pyelonephritis, bacteremia and urosepsis. Acute pyelonephritis often requires hospitalization and when accompanied by bacteremia, acute pyelonephritis has a mortality rate of 10% to 20% [3]. Urosepsis is responsible for 25% of all sepsis cases and sepsis is a life-threatening infection that must be treated instantly [4,5]. Every year approximately 30 million people are afflicted by sepsis, which has a mortality rate of 30–40% [6,7]. Rapid diagnosis and suitable treatment are crucial during sepsis, as the risk of mortality rises for each hour without adequate treatment [6,7]. Therefore, we need a better understanding of how UPEC bacteria modulate the immune responses in the kidney and bloodstream, in order to prevent bacterial infiltration into the bloodstream from the kidneys. This knowledge could help us prevent the onset of urosepsis and reduce mortality. 

Fibroblasts have historically been perceived as structural cells that synthesize and regulate the extracellular matrix in tissues [8,9]. However, we and others have shown that fibroblasts are important immunoreactive cells that can recognize pathogens and induce chemokines and cytokines to recruit leukocytes to the infection site [8,9,10,11]. After penetrating through the renal epithelium, UPEC are in direct contact with interstitial renal fibroblasts before reaching the bloodstream. The result of this interaction is largely unknown. We have previously shown that primary renal fibroblasts are strong immunoreactive cells that have the same capacity as renal epithelial cells to recruit neutrophils in response to a UPEC infection in vitro [11]. 

Inflammasomes are cytosolic multiprotein complexes that detect bacterial virulence factors and/or danger/damage signals and trigger an immune response by the maturation and release of proinflammatory cytokines such as IL-1β and IL-18 and induce pyroptosis (lytic cell death). When the inflammasome assembles, it can activate proinflammatory caspases in the cytosol, such as caspase-1 and caspase-4, which convert pro-IL-1β into active IL-1β [12]. Furthermore, human caspase-4/-5 were recently reported to be cytosolic receptors for LPS [13,14], which is interesting as UPEC is known to invade both epithelial cells and fibroblasts [11,15,16]. It has recently been shown that UPEC can activate the nod like receptor pyrin domain containing 3 (NLRP3) inflammasome in neutrophils and bladder epithelial cells [17,18,19]. Although it has been highlighted in recent years that the NLRP3 inflammasome plays an important role during a UPEC mediated UTI [20], studies showed contradictory results. Some publications have shown that IL-1β has a role in the immune response that limits the colonization of the urinary tract [21,22]. Another study found that IL-1β knockout mice were protected from developing UPEC mediated UTI, which indicates that IL-1β may drive the infection [23]. Presently there is limited knowledge on the role of renal fibroblasts in the release of IL-1β from the kidney during a UTI. We know that UPEC can induce IL-1β release from bladder epithelial cells and neutrophils [17,19], but not from renal epithelial cells [23]. This suggests a specificity for the bladder epithelium and infiltrating neutrophils. The aim of this study was to investigate if renal fibroblasts can release IL-1β during a UPEC infection and if so, to elucidate the mechanism behind the UPEC mediated IL-1β release. 

## 2. Methods 

### 2.1. Cell and Bacterial Culture

Human renal medullary fibroblast cell line TK173 [24] (kind gift from Professor Anton Jan van Zonneveld, Leiden University, Leiden, Netherlands), human renal epithelial cell line A498 (HTB-44, American Type Culture Collection (ATCC), Manassas, VA, USA) and the human immortalized proximal tubule epithelial cell line HK-2 (ATCC) were used in this study. All three cell lines were cultured in Dulbecco’s modified eagle medium (DMEM, Lonza, Basel, Switzerland) containing 10% fetal bovine serum (FBS), 2 mM L-glutamine, 1 mM non-essential amino acids (Thermo fisher Scientific, Waltham, MA, USA) at 37 °C in a 5% CO_2_ atmosphere. The cells were serum starved overnight in DMEM containing 2 mM L-glutamine, 1 mM non-essential amino acids. During experiments, DMEM containing 1% FBS, 1 mM non-essential amino acids and 2 mM l-glutamine were used. CFT073 is a UPEC isolated from a patient with pyelonephritis and urosepsis [25]. Prior to experiments, CFT073 and its mutants [17,26] was grown in Lysogeny broth (Difco Laboratories, Detroit, MI, USA) overnight on shake at 150 rpm at 37 °C.

### 2.2. CRISPR/Cas9 Genome Editing

CRISPR/Cas9 gene editing in the renal fibroblasts was conducted using the pSpCas9 (BB)-2A-Puro (PX459, V2.0) (a gift from Feng Zhang, Addgene plasmid # 62988) [27] plasmid. Plasmid transfection was performed using Lipofectamine 2000 (Life Technologies, Carslbad, CA, USA). The target sites were: GCTAATGATCGACTTCAATG (NLRP3), TGCAGCTCATCCGAATATGG (Caspase-4) and GACAGTATTCCTAGAAGAAC (Caspase-1). The renal fibroblasts were selected with pyromycin (2.5 µg/mL, Sigma-Aldrich, St. Louis, MO, USA) 24 h after transfection. All experiments were performed with a polyclonal pool of gene-edited cells. The gene editing was confirmed by Western Blot analysis, as previously described [28].

### 2.3. Stimulation of Renal Fibroblasts and Renal Epithelial Cells

The renal fibroblasts and the renal epithelial cell lines HK-2 and A498 cells were stimulated with CFT073 wild-type and mutants at a multiplicity of infection (MOI) of 1 or 10 for 6 h and incubated at 37 °C with 5% CO_2_. The fibroblasts were also pre-incubated with DMSO (vehicle), p38 MAPK inhibitor SB203580 (10 µM, Santa Cruz Biotechnology Inc., Heidelberg, Germany), ERK1/2 inhibitor PD98059 (10 µM, Santa Cruz Biotechnology Inc.), NF-κB inhibitor BAY 11-7082 (5 µM, Enzo Life Sciences, Farmingdale, NY, USA) and the PKC inhibitor bisindolylmaleimide I (10 µM, Santa Cruz Biotechnology Inc.), JNK inhibitor SP600125 (10 μM, InSolutionT M JNK Inhibitor II, Calbiochem, San Diego, CA, USA), serine protease inhibitor 3,4-Dichloroisocoumarin (DCI, 100 μM, Merck Millipore, MA, USA), cathepsin B inhibitor CA074 (100 μM, Apexbio Technology LLC, Houston, TX, USA), NLRP3 inhibitor MCC950 (2 μM, Avistron Chemistry Services, Cornwall, UK), Disulfiram (10 μM, Selleckchem, Houston, TX, USA), caspase-3 inhibitor AC-DEVD-CHO (10 μM, Enzo Life Sciences) for 1 h prior to CFT073 stimulation for 4 or 6 h at MOI 1. Supernatants and total RNA were collected and kept at −80 °C until further analysis.

### 2.4. RNA Isolation and Real Time RT-PCR

Total RNA from renal fibroblasts and the renal epithelial cell lines HK-2 and A498 cells were isolated using the E.Z.N.A. Total RNA Kit I (Omega, Bio-Tek, Norcross, GA, USA). Quantification of the RNA yield was performed using the Nano-Drop ND-1000 spectrophotometer (Wilmington, NC, USA). An amount of 100 ng total RNA was used to synthesize a first strand cDNA with the High-capacity cDNA RT kit (Thermo Fisher Scientific, Waltham, MA, USA). The real time RT-PCR was conducted using 10 ng cDNA, Maxima SYBR Green qPCR Master Mix (Thermo Fisher Scientific) and 250 nM of each primer (Table 1). The primers were designed by Origene (Rockville, MD, USA) and synthesized by Eurofins MWG Synthesis GmbH (Munich, Germany). The PCR was performed using the CFX96 Touch Real-Time PCR Detection System (Bio-Rad Laboratories, Hercules, CA, USA). The procedure used was: desaturation at 95 °C for 10 min, 40 cycles of denaturation at 95 °C for 15 s and finally annealing/extension at 60 °C for 60 s. The mRNA expression was assessed by the comparative Ct (ΔΔCt) method followed by normalization to the endogenous control GAPDH. Fold difference was calculated as 2^−ΔΔCt^.

### 2.5. Measurement of IL-1β and LDH Release

Supernatants were collected after bacterial infection of renal fibroblasts and renal epithelial cells and stored at −80 °C. An enzyme-linked immunosorbent assay (ELISA) was performed to measure IL-1β release (ELISA MAX™ Deluxe Sets, BioLegend, San Diego, CA, USA) according to the manufacturer’s instructions. Cell viability was evaluated by Pierce Lactate dehydrogenase (LDH) cytotoxicity assay (Thermo Fisher Scientific) according to the manufacturer’s instructions. Both assays were evaluated using the Cytation 3 plate reader (BioTek, Winooski, VT, USA), as previously described [28].

### 2.6. Caspase-1 and Caspase-4 Activity Assay

Renal fibroblasts were cultivated in a 96-well plate and pre-incubated with the caspase-1 substrate Ac-YVAD-AMC (Enzo Life Sciences, Farmingdale, NY, USA) or the caspase-4 substrate Ac-LEVD-AMC (Enzo Life Sciences, Farmingdale, NY, USA) for 1 h at 37 °C 5% CO_2_. The fibroblasts were infected with CFT073 at MOI 1 and analyzed after 4 and 6 h with the Cytation 3 (BioTek) plate reader at excitation/emission settings of 340/440 nm.

### 2.7. Western Blot Analysis

The renal fibroblasts were lysed in radioimmunoprecipitation assay (RIPA) buffer complemented with a phosphatase inhibitor mix (Thermo Fisher Scientific). Protein quantification was performed using the DC protein assay (Bio-Rad Laboratories, Hercules, CA, USA). The proteins were mixed with Laemmli buffer, boiled for 5 min at 95 °C and separated on a 4–15% SDS-polyacrylamide gel electrophoresis and transferred onto a polyvinylidene fluoride membrane (PVDF) (Bio-Rad Laboratories). Three percent BSA in Tris-buffered Saline 0.1% Tween 20 (TBST) was used to block the PVDF membrane for 1 h at room temperature. The membrane was incubated overnight at 4 °C with the primary antibodies. Human caspase-1 was detected using a rabbit polyclonal antibody (Santa Cruz Biotechnology, Dallas, TX, USA). Human caspase-4 was detected using a rabbit monoclonal antibody (Cell signaling Technologies, Danvers, MA, USA) and human NLRP3 was detected by using a rabbit monoclonal antibody (Cell signaling Technologies). GAPDH was detected by using a rabbit polyclonal antibody (Santa Cruz Biotechnology). As secondary antibodies, goat anti-rabbit IgG (HRP) (Santa Cruz Biotechnology) and goat anti-mouse IgG (HRP) (Santa Cruz Biotechnology) were used and incubated for 1 h at room temperature. The membrane was imaged using Luminata Forte Western HRP Substrate (Merck Millipore, Darmstadt, Germany), as previously described [28]. 

### 2.8. Immunofluorescence

The renal fibroblasts were infected with CFT073 at MOI 1 for 4 h and incubated at 37 °C with 5% CO_2_. After infection, the cells were washed with PBS and fixed for 15 min in 4% paraformaldehyde. Cells were then permeabilized using 0.1% Triton X-100 in PBS for 10 min. Cells were incubated with 1% bovine serum albumin (BSA) for 30 min to block unspecific antibody binding. Human NLRP3 was detected by using a mouse monoclonal anti-NLRP3 antibody (Abnova, Taipei, Taiwan), diluted 1:50, for 1 h (in PBS with 1% BSA). Human ASC was detected by using a rabbit polyclonal anti-ASC antibody (Santa Cruz Biotechnology), diluted 1:50, for 1 h (in PBS with 1% BSA). For NLRP3, a secondary goat polyclonal anti-mouse A488 conjugated antibody (Abcam), diluted 1:1000, was used for 1 h (in PBS with 1% BSA). For ASC, a secondary goat polyclonal anti-rabbit A594 conjugated antibody (Thermo Fisher Scientific), diluted 1:1000, was used for 1 h (in PBS with 1% BSA). The nucleus was stained using 4′, 6-diamidino-2-phenylindole (DAPI; Santa Cruz Biotechnology) for 10 min. Samples were evaluated with the Olympus BX53 microscope (Olympus, Tokyo, Japan), as previously described [24]. 

### 2.9. Conditioned Medium

The Cas9, caspase-1, caspase-4 and NLRP3-deficient renal fibroblasts were infected with CFT073 for 6 h at MOI 1 and incubated at 37 °C with 5% CO_2_. Supernatants were centrifuged at 5000× *g* for 5 min to remove the CFT073 bacteria and then frozen at −80 °C. These supernatants were defined as conditioned medium. TSA agar plating was performed to exclude the presence of viable bacteria in the conditioned medium.

### 2.10. Neutrophil Isolation

Density gradient centrifugation of polymorphprep and lymphoprep reagents (AXIS-SHIELD PoC AS, Oslo, Norway) was used to isolate human neutrophils from healthy blood donors according to the manufacturers. Neutrophil viability was >90% as determined by Trypan blue. An ethical approval was granted by the regional ethics review board in Uppsala, Sweden (Dnr 2015/437), to isolate blood from healthy individuals after informed consent. Blood from healthy donors was collected according to the ethical guidelines of both the Declaration of Helsinki and the Swedish national board of health and welfare. 

### 2.11. Measurement of Reactive Oxygen Species (ROS)

Total reactive oxygen species (ROS) production from neutrophils was measured using a luminol-horseradish peroxidase (HRP) assay. The neutrophils were incubated with HRP (4 U/mL, Roche) and luminol (0.1 mg/mL, Sigma) for 15 min in 5% CO_2_ at 37 °C. The neutrophils (10^6^) were added to a 96-well plate with respective conditioned medium from the infected renal fibroblasts (cas9, caspase-1, caspase-4 and NLRP3). The luminescence was measured with the Cytation 3 plate reader after 20 min, as previously described [19]. 

### 2.12. Phagocytosis Assay

Conditioned medium from caspase-1, caspase-4 and NLRP3-deficient renal fibroblasts was added to a 96-well plate and neutrophils (200,000 cells) were then added to the respective wells. CFT073 (carrying an eGFP-plasmid) at MOI 10 was used to infect the conditioned medium and neutrophils for 3 h. Neutrophils were washed twice with PBS to remove non-phagocytosed CFT073 and 0.2% Trypan blue (Thermo fisher Scientific) was then added to the cells to quench the remaining eGFP signal from extracellular CFT073. Samples were acquired to measure mean florescence intensity of phagocytized CFT073 (eGFP) bacteria using the Gallius (Beckman Coulter, Brea, CA, USA) flow cytometer with a 488 nm laser and an FL1 525/40 nm band-pass filter. The data were analyzed using Kaluza Flow Cytometry Analysis v1.3 (Beckman Coulter), as previously described [19]. 

### 2.13. Statistical Analysis

The differences between groups were analyzed with one-way ANOVA followed by a Bonferroni test. Differences were considered statistically significant when *p* < 0.05. Data are presented as mean ± SEM, where *n* = the number of independent experiments. 

## 3. Results

### 3.1. IL-1β and LDH Release from Renal Fibroblasts and Renal Epithelial Cells

**Figure 1:**
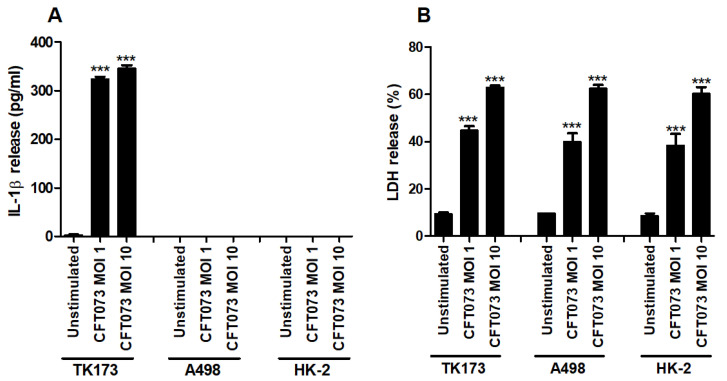
IL-1β and LDH release during a UPEC infection. Renal fibroblasts (TK173) and renal epithelial cells (HK-2 and A498) were infected with UPEC strain CFT073 at MOI 1 or MOI 10 for 6 h followed by analysis of IL-1β (**A**) and LDH release (**B**). Data are presented as mean ± SEM (*n* = 3 independent experiments). Asterisks over bars denote statistical significance compared to respective unstimulated control (*** *p* < 0.001).

The human renal fibroblast cell line TK173, the human renal epithelial cell line A498 and the human immortalized proximal tubule epithelial cell line HK-2 were infected with CFT073 and the release of IL-1β and LDH was assessed. We found that CFT073 significantly increased the release of IL-1β from renal fibroblasts at MOI 1 and 10 after 6 h of infection compared to unstimulated cells (Figure 1A). No basal or CFT073-induced IL-1β release was observed from either of the renal epithelial cell lines after 6 h. CFT073 induced significantly increased LDH release from both renal fibroblasts and renal epithelial cells compared to unstimulated cells. However, CFT073-induced LDH release was not different between fibroblasts and epithelial cells (Figure 1B). Taken together, these findings show that renal fibroblasts, but not renal epithelial cells, can induce IL-1β release upon a UPEC infection.

### 3.2. The UPEC-Strain CFT073 Induces Inflammasome Activation

**Figure 2:**
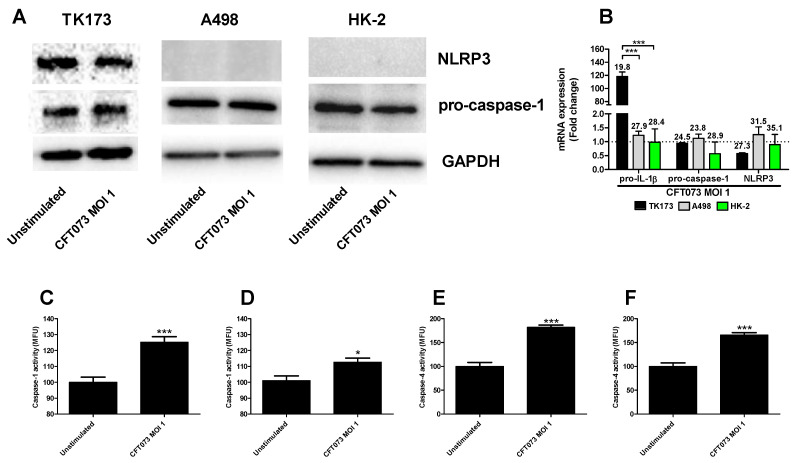
Caspase-1 and caspase-4 activity and pro-IL-1β, pro-caspase-1 and NLRP3 expression. Renal fibroblasts (**A**–**F**) and renal epithelial cells (**A**,**B**) were infected with UPEC strain CFT073 at MOI 1 for 4 (**A**–**C**,**E**) and 6 h (**D**,**F**) followed by analysis of pro-caspase-1 and NLRP3 protein expression by Western blot (**A**), gene expression (**B**) and caspase-1 (**C**,**D**) and caspase-4 activity (**E**,**F**). Caspase-1/4 activity is presented as fold increase of mean fluorescence units (MFU) compared to unstimulated cells. GAPDH was used as a loading control for Western blots. The dotted line represents the unstimulated cells. Data are presented as mean ± SEM (*n* = 3 independent experiments). Asterisks over bars denote statistical significance compared to respective unstimulated control, * *p* < 0.05, *** *p* < 0.001).

**Figure 3:**
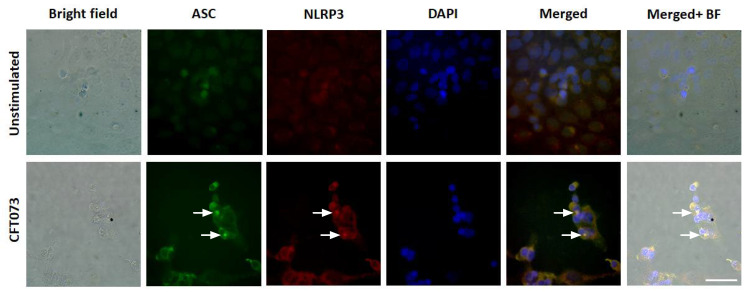
ASC-speck formation during a UPEC infection. Renal fibroblasts were infected with UPEC strain CFT073 at MOI 1 for 4 and ASC-speck formation and NLRP3 localization were visualized with fluorescence microscopy. Green represents ASC, red represents NLRP3 and blue (DAPI) represents the nucleus. Scale bar represents 50 µm. Arrows show colocalized specks. Images are representative of 3 independent experiments.

We continued to investigate the mechanisms behind the differences in CFT073 mediated IL-1β release from renal fibroblast and renal epithelial cells. We found that NLRP3 and caspase-1 are constitutively expressed at the protein level in renal fibroblasts (Figure 2A). However, only caspase-1 was found to be expressed in the renal epithelial cells and not NLRP3 (Figure 2A) at the basal level. This was also validated by real time PCR showing a mean Ct-value of 27.3 (Renal fibroblasts), 31.5 (A498) and 35.1 (HK-2) for NLRP3 after CFT073 infection at MOI 1 for 4 h (Figure 2B). We also found that renal fibroblasts, but not renal epithelial cells, induced a significantly increased gene expression of pro-IL-1β (120-fold) after 4 h of CFT073 infection at MOI 1 compared to unstimulated cells (Figure 2B). We then moved on to elucidate if caspase-1 and caspase-4 were activated in renal fibroblasts by CFT073. We found that CFT073 at MOI 1 induced a significant caspase-1 activation compared to unstimulated cells after 4 (Figure 2C) and 6 h (Figure 2D). We found the same to be true for caspase-4 activation after 4 (Figure 2E) and 6 h (Figure 2F) of CFT073 infection. Using immunofluorescence staining of ASC and NLRP3, we found that CFT073 at MOI 1 after 4 h on infection induced ASC-speck formation and that these specks were co-localized with NLRP3 in renal fibroblasts (Figure 3). Our findings show that the UPEC strain CFT073 can induce the activation of the NLRP3 inflammasome in renal fibroblasts.

### 3.3. Signaling Pathways Associated with CFT073 Mediated IL-1β and LDH Release

**Figure 4:**
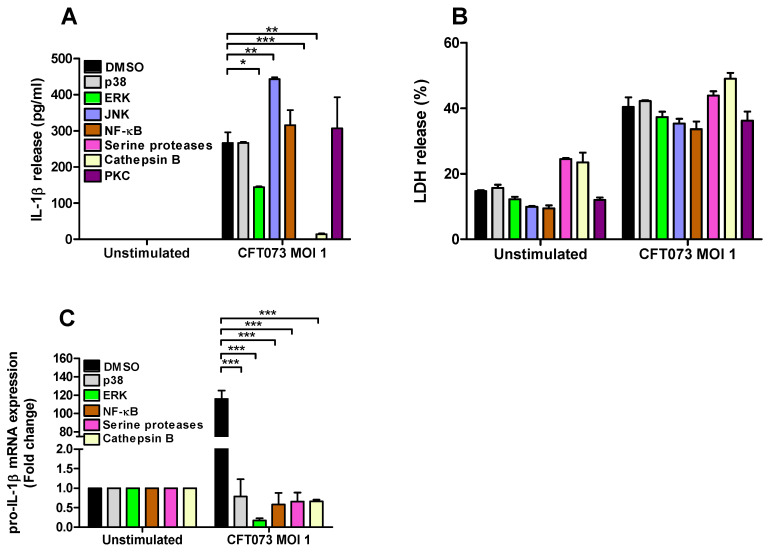
Signaling pathways associated with UPEC-induced pro-IL-1 β expression, IL-1β and LDH release. Renal fibroblasts were pre-incubated with DMSO (vehicle), JNK inhibitor SP600125 (10 µM), p38 MAPK inhibitor SB203580 (10 µM), ERK1/2 inhibitor PD98059 (10 µM), NF-κB inhibitor BAY 11-7082 (5 µM), serine protease inhibitor 3,4-Dichloroisocoumarin (DCI, 100 μM), cathepsin B inhibitor CA074 (100 μM) and PKC inhibitor bisindolylmaleimide I (10 µM (**A**,**B**) for 1 h prior to infection with CFT073 at MOI 1 for 4 (**C**) or 6 h (**A**,**B**) followed by analysis of IL-1β (**A**) and LDH release (**B**) and the gene expression of pro-IL-1 β (**C**). Data are presented as mean ± SEM (*n* = 3 independent experiments) (* *p* < 0.05, ** *p* < 0.01, *** *p* < 0.001).

To assess the signaling pathways involved in CFT073 mediated IL-1β and LDH release and pro-IL-1β expression from renal fibroblasts inhibitors targeting p38, ERK1/2, JNK, NF-κB, serine proteases, cathepsin B and PKC were utilized. We found that inhibition of ERK1/2, serine proteases and cathepsin B, but not p38, NF-κB and PKC, resulted in significantly lower IL-1β release compared to DMSO treated cells after CFT073 infection at MOI 1 for 6h. In addition, we also found that inhibiting JNK increased the release of IL-1β after CFT073 infection (Figure 4A). We did not find any significant differences in LDH release after CFT073 infection at MOI 1 for 6h compared to DMSO treated cells using the respective signaling inhibitors (Figure 4B). In addition, we found that inhibition of p38, ERK, NF-κB, serine proteases and Cathepsin B resulted in significantly lower expression of pro-IL-1β compared to DMSO treated cells after CFT073 infection at MOI 1 for 4h (Figure 4C).

### 3.4. α-Hemolysin Mediates IL-1β Release from Renal Fibroblasts

**Figure 5:**
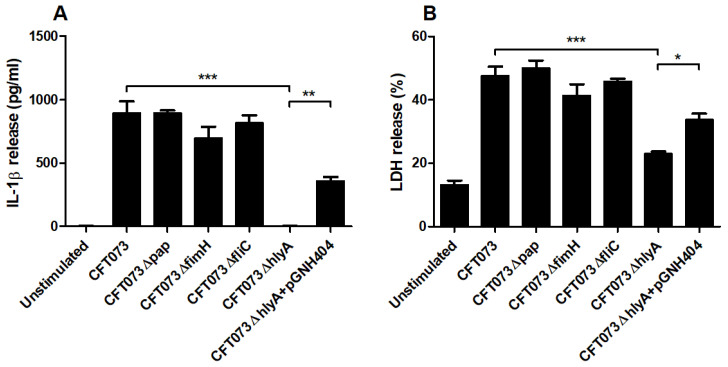
UPEC virulence factors associated with IL-1β and LDH release. Renal fibroblasts were infected with UPEC strain CFT073, CFT073Δ*pap*, CFT073Δ*fimH*, CFT073Δ*fliC*, CFT073Δ*hlyA* or CFT073ΔhlyA/pGNH404 at MOI 1 for 6 h followed by analysis of IL-1β (**A**) and LDH (**B**) release. Data are presented as mean ± SEM (*n* = 3 independent experiments) (* *p* < 0.05, ** *p* < 0.01, *** *p* < 0.001).

We proceeded with evaluating the involvement of various UPEC virulence factors on IL-1β release using P-fimbriae (*pap*), type-1 fimbriae (*fimH*), Flagellin (*fliC*) and α-hemolysin (*hlyA*) CFT073 deletion mutants. We found that Δ*hlyA* induced a significantly lower IL-1β release compared to the wild-type CFT073 (Figure 5A). We also showed that the α-hemolysin complemented CFT073ΔhlyA/pGNH404 strain induced significantly higher IL-1β release compared to Δ*hlyA*. However, the Δ*pap*, Δ*fliC* and Δ*fimH* deletion mutants did not show any differences in IL-1β release compared to the wild-type CFT073 (Figure 5A). Furthermore, we also found that Δ*hlyA*, but not Δ*pap*, Δ*fliC* and Δ*fimH*, induced a significantly lower LDH release compared to the wild-type CFT073 and the α-hemolysin complemented CFT073ΔhlyA/pGNH404 strain. These data suggest that IL-1β and LDH release from renal fibroblasts during a UPEC infection are α-hemolysin dependent.

### 3.5. Involvement of Caspase-1, Caspase-4, NLRP3 and Gasdermin D in the Release of IL-1β and LDH

**Figure 6:**
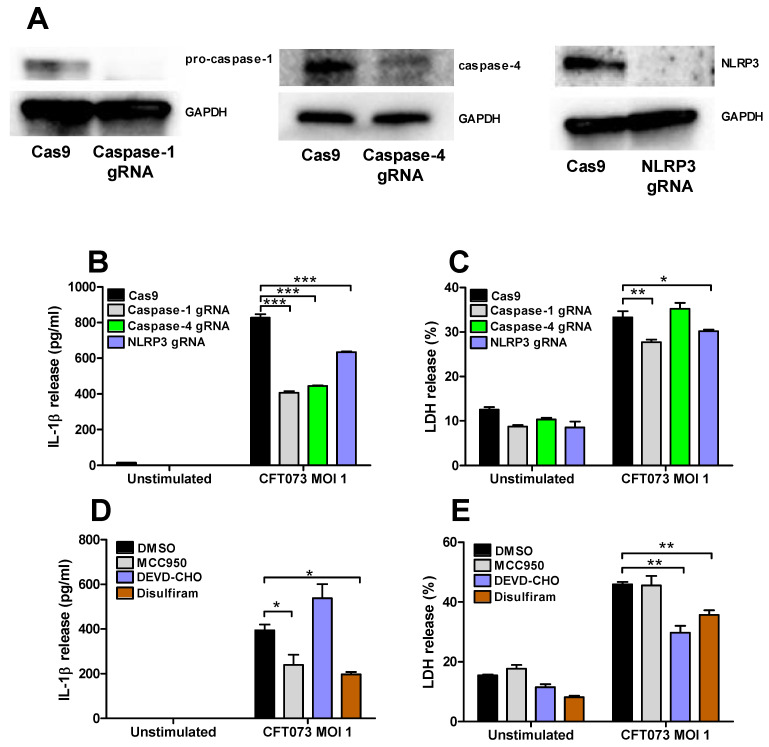
Inflammasome-associated proteins role in IL-1β and LDH release. The knockdown of caspase-1, caspase-4 and NLRP3 in renal fibroblasts cells using the CRISPR/Cas9 system was evaluated by Western blot analysis. GAPDH was used as the loading control (**A**). IL-1β (**B**) and LDH (**C**) release from Cas9, caspase-1, caspase-4 or NLRP3-deficient renal fibroblasts after infection with UPEC strain CFT073 at MOI 1 for 6 h. Renal fibroblasts were pre-incubated with DMSO (vehicle), caspase-3 inhibitor Ac-DEVD-CHO (10 μM), NLRP3 inhibitor MCC950 (2 μM) or the Gasdermin D inhibitor Disulfiram (10 μM) for 1 h prior to infection with CFT073 at MOI 1 for 6 h followed by analysis of IL-1β (**D**) and LDH (**E**) release. Data are presented as mean ± SEM of 3 independent experiments (* *p* < 0.05, ** *p* < 0.01, *** *p* < 0.001).

Renal fibroblasts cells deficient of caspase-1, caspase-4 and NLRP3 were constructed with the CRISPR/Cas9 system. Western blot evaluation confirmed a decreased protein expression of caspase-1, caspase-4 and NLRP3 compared to renal fibroblasts transfected with a control Cas9 plasmid (Figure 6A). The deficient fibroblasts were then infected with CFT073 at MOI 1 for 6 h and IL-1β and LDH release were evaluated. CFT073 induced a significantly lower IL-1β release from caspase-1, caspase-4 and NLRP3-deficient cells compared to the CFT073 stimulated Cas9 control cells (Figure 6B). However, only caspase-1 and NLRP3-deficient cells induced a significantly lower LDH release compared to the CFT073 stimulated Cas9 control cells (Figure 6C). We also found that the NLRP3 inhibitor MCC950 and the Gasdermin D inhibitor Disulfiram significantly reduced CFT073 induced IL-1β release compared to DMSO treated cells (Figure 6D). However, only the caspase-3 inhibitor Ac-DEVD-CHO and Gasdermin D inhibitor Disulfiram, but not the NLRP3 inhibitor MCC950, significantly reduced the CFT073 induced LDH release compared to DMSO treated cells (Figure 6E).

### 3.6. Phagocytosis and ROS-Production

**Figure 7:**
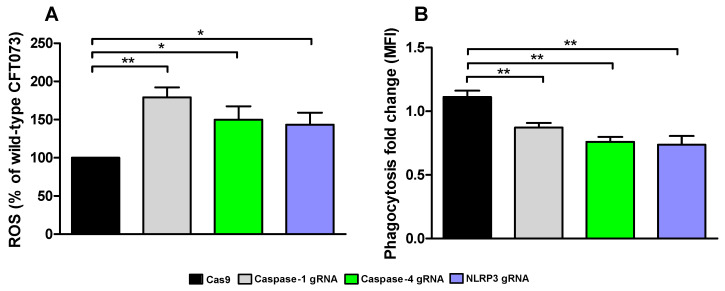
UPEC phagocytosis and production of reactive oxygen species (ROS). Neutrophils were subjected to conditioned medium from Cas9 controls, caspase-1, caspase-4 and NLRP3-deficient fibroblasts and ROS-production (**A**) was measured after 20 min and phagocytosis (**B**) of CFT073 (eGFP) was evaluated after 3 h. Phagocytosis is presented as fold change (mean florescence intensity) relative to the unstimulated control. ROS is presented as % of wild-type CFT073. Data are presented as mean ± SEM of 3 independent experiments (* *p* < 0.05, ** *p* < 0.01).

We continued with investigating how conditioned medium from the caspase-1, caspase-4 and NLRP3-deficient cells affected ROS-production and phagocytosis by human neutrophils. We found that conditioned medium from caspase-1, caspase-4 and NLRP3-deficient cells induced a significantly increased ROS-production from neutrophils compared to conditioned medium from CFT073 stimulated Cas9 control cells (Figure 7A). We also found that conditioned medium from caspase-1, caspase-4 and NLRP3-deficient cells significantly decreased phagocytosis of CFT073 compared to Cas9 control cells (Figure 7B). These data suggest that the renal fibroblast milieu mediated by inflammasome-related proteins can modulate the antimicrobial phenotype of neutrophils.

## 4. Discussion

Understanding the pathological mechanisms by which UPEC modulates the immune response in the kidney is essential to prevent UPEC from reaching the bloodstream and causing urosepsis. The research has so far been focused on investigating the interaction of UPEC with infiltrating leukocytes and renal epithelial cells [19,29,30,31]. However, the role of renal fibroblasts in the progression of a UTI is not well known. The source of IL-1β during a UTI has mainly been associated with activation of the NLRP3 inflammasome in bladder epithelial cells, macrophages and neutrophils [17,18,19,32], but less is known about the contribution of renal fibroblasts. Our aim for this study was to investigate if renal fibroblasts can release IL-1β during a UPEC infection. 

We started by evaluating if CFT073 could induce the release of IL-1β and LDH from renal fibroblasts and renal epithelial cells. We found that only the renal fibroblasts induced a significantly increased IL-1β release upon a UPEC infection. Interestingly, neither the A498 nor the HK-2 renal epithelial cell line released IL-1β at a basal level or after a UPEC infection. However, the IL-1β associated LDH release was found to be increased in both the renal fibroblasts and renal epithelial cells. Others have also observed that renal epithelial cells do not release IL-1β during a UPEC infection [23]. The renal epithelial cells did not have any basal or UPEC induced expression of NLRP3, which was validated by Western blot and real time PCR. We also found that UPEC was only able to induce an increased gene expression of pro-IL-1β from renal fibroblasts and not from renal epithelial cells. These findings may explain why renal epithelial cells do not release IL-1β during a UPEC infection. Others have found that the renal epithelial cell line HK-2 can express NLRP3 [33,34], but this does not seem to be true during a UPEC infection. To our knowledge this is the first study demonstrating that the source of IL-1β has been linked to renal fibroblasts during a UPEC infection. One may contemplate why renal epithelial cells do not release IL-1β, but bladder epithelial cells do? Is the initial IL-1β release from the bladder epithelial cells enough to fight of the infection? Would the release of IL-1β from renal epithelial cells just aggravate the infection? Are renal fibroblasts checkpoint cells that release IL-1β to alert the immune system that UPEC has breached the renal epithelial cell layer? Some studies have found that IL-1β limits the UPEC colonization of the urinary tract [21,22]. However, another study found that IL-1β-deficient mice were protected from developing UPEC mediated UTI, which suggests that IL-1β may drive the infection [23]. Understanding the selectiveness of IL-1β release from the urinary tract may help us understand IL-1β’s role during a UPEC mediated UTI. 

We proceeded with evaluating if the IL-1β release was associated with NLRP3 inflammasome assembly, caspase-1 and caspase-4 activation and pyroptosis. We observed a UPEC mediated caspase-1 and caspase-4 activation and ASC-speck formation in renal fibroblasts. NLRP3 was also found to be colocalized to the ASC-specks. We also found that the release of IL-1β was significantly reduced in caspase-1, caspase-4 and NLRP3-deficient cells compared to CFT073 stimulated Cas9 cells. In addition, inhibiting NLRP3, Gasdermin D, but not caspase-3, also reduced the IL-1β release upon a UPEC infection. Activation of the NLRP3 inflammasome, caspase-1 and caspase-4 are known to lead to the maturation and release of IL-1β in several cell types including fibroblasts [13,17,18,19,35]. However, we only observed a small reduction in LDH release from the caspase-1 and NLRP3-deficient cells, the Gasdermin D inhibitor and the caspase-3 inhibitor. Hence, it seems that the inflammasome mediated IL-1β release from renal fibroblasts upon a UPEC infection takes place partly independent of pyroptosis and apoptosis. In agreement with our findings, it has been shown that NLRP3 activation in neutrophils occurs independent of lytic cell death [19,36]. 

We then continued with investigating the signaling pathways associated with IL-1β release from renal fibroblasts. We revealed that the release of IL-1β from renal fibroblasts was dependent on ERK 1/2 and cathepsin B, but not NF-κB, p38, PKC or JNK upon a UPEC infection. We also found that the increased gene expression of pro-IL-1β in renal fibroblasts was dependant on p38, ERK 1/2, NF-κB, serine proteases and cathepsin B during a UPEC infection. The activation and assembly of the NLRP3 inflammasome is recognized to be dependent on priming and induction of signaling pathways that induces gene expression and post-translational modifications of NLRP3 associated inflammasome proteins [37]. At first, inflammasome priming was assumed to be mediated through a TIR/MyD88/NF-κB-dependent manner [38], but we know now that the priming cascade is regulated differently depending on cell type and stimuli. The signaling molecules ERK 1/2 [39], p38 [40], PKC [41] and NF-κB [37] have all been found to be engaged in NLRP3 inflammasome priming. In addition, cathepsin B leakage from lysosomes has also been linked to NLRP3 inflammasome activation [37]. Interestingly, p38 and NF-κB seem to be more involved in the gene induction of pro-IL-1β, rather than the release of IL-1β. This indicates that basal expression of pro-IL-1β in renal fibroblasts is sufficient for UPEC to induce a maturation and release of IL-1β. Based on our Western blot and real time PCR data, the NLRP3 and pro-caspase-1 proteins were detected in unstimulated fibroblasts, suggesting a constitutive expression of the NLRP3 inflammasome pathway in renal fibroblasts. Furthermore, as the caspase-1, caspase-4 and NLRP3-deficient cell lines still had the capacity to release IL-1β, we continued to investigate if additional enzymes could be involved in IL-1β release. We and others have shown that serine proteases are involved in the maturation and release of IL-1β from neutrophils and bladder epithelial cells after a UPEC infection [17,42,43]. We showed, using a serine protease inhibitor, that IL-1β release was completely inhibited. It has previously been shown that the UPEC virulence factor α-hemolysin can activate the serine protease mesotrypsin in bladder epithelial cells. This activation triggered proteolysis of host proteins and subsequent cell exfoliation [44]. We have previously shown that mesotrypsin is involved in the IL-1β release from bladder epithelial cells upon a UPEC infection [17]. This suggests that mesotrypsin may be involved in the UPEC mediated release of IL-1β from renal fibroblasts also. Interestingly, neither ERK 1/2, cathepsin B nor serine protease inhibitors altered the UPEC mediated LDH release, thereby strengthening the notion that IL-1β release from renal fibroblasts upon a UPEC infection takes place partly independent of pyroptosis. Taken together, our findings suggest that the release of IL-1β from renal fibroblasts, mediated by UPEC infection, involves ERK 1/2, cathepsin B and serine proteases.

UPEC is known to harbor several virulence factors that can alter the host immune response to promote colonization of the urinary tract [45,46]. However, the interaction of UPEC virulence factors with renal fibroblasts in terms of IL-1β release is not known. Using type-1 fimbriae (*fimH*), p-fimbriae (*pap*), flagellin (*fliC*) and α-hemolysin (*hlyA*) deletion mutants we found that only the Δ*hlyA* deletion mutant induced a significantly lower IL-1β and LDH release compared to the wild-type UPEC strain. Furthermore, we also showed that the α-hemolysin complemented CFT073ΔhlyA/pGNH404 strain restored the IL-1β and LDH release. The lytic cell death mediated by the pore forming toxin α-hemolysin may explain the IL-1β release that is not associated with pyroptosis or apoptosis during a UPEC infection of renal fibroblasts. α-hemolysin is expressed in approximately 50% of pyelonephritis and 40% of cystitis isolates [47], and α-hemolysin is known to have several effects on human cells. At a low concentration, α-hemolysin is immunomodulatory and at a high concentration it induces lytic cell death [48]. We have previously found that α-hemolysin, but not p-fimbriae or type-1 fimbriae expression, is essential for NLRP3 activation and IL-1β release from human bladder epithelial cells during a UPEC infection [17]. Taken together, our findings have shown that α-hemolysin may be the key UPEC virulence factor for mediating IL-1β release from renal fibroblasts during a UPEC infection.

An essential part of the immune response to a UPEC infection is the infiltration of neutrophils to the urinary tract [49]. During a UTI, neutrophils are vital for the clearance of the infection. They kill UPEC by ROS, phagocytosis, neutrophil extra cellular traps and by the release of antimicrobial peptides [50,51,52,53,54]. Therefore, we wanted to elucidate what role the caspase-1, caspase-4 and NLRP3 mediated milieu from renal fibroblasts play in ROS-production and phagocytosis. We found that conditioned medium from caspase-1, caspase-4 and NLRP3-deficient renal fibroblasts evoked significantly increased ROS-production from neutrophils. In addition, we also observed that phagocytosis of CFT073 by neutrophils was decreased in conditioned medium from UPEC-infected caspase-1, caspase-4 and NLRP3-deficient cells. UPEC has been shown to be able to suppress NLRP3 by the virulence factor TcpC [21] and thus able to create an NLRP3-deficent milieu in patients. The inflammasome associated proteins NLRP3, caspase-1 and caspase-4 have been shown by us [unpublished data] and others [55,56,57,58,59] to be involved in the regulation and release of several important neutrophil activators and modulators such as IL-1β, IL-6, IL-8, IL-17C, LIF etc. We hypothesize that it is not a single meditator, but a mixture of stimuli that promote the observed changes induced by the conditioned medium from renal fibroblasts. Hence, when the neutrophils infiltrate, the renal milieu may promote a ROS-producing, rather than a phagocytic neutrophil phenotype during a UPEC infection. However, the impact this phenotypical shift has on the infection needs further investigation. 

In conclusion, our findings show that renal fibroblasts, but not renal epithelial cells release IL-1β during a UPEC infection. The IL-1β release was observed to be NLRP3 inflammasome, serine protease and α-hemolysin dependent. Interestingly, the IL-1β release was not fully associated with pyroptosis. This study sheds light on the role of renal fibroblasts as orchestrators of immune responses trying to prevent UPEC from reaching the bloodstream, which was previously undermined. 

## Figures and Tables

**Table 1:** Primers used for quantitative real-time PCR.

Gene Symbol	Oligonucleotide Sequences (5′-3′)
*pro-IL-1β*	*F:* CCACAGACCTTCCAGGAGAATG *R:* GTGCAGTTCAGTGATCGTACAGG
*P*ro-*Caspase-1*	*F:* GCTGAGGTTGACATCACAGGCA *R:* TGCTGTCAGAGGTCTTGTGCTC
*NLRP3*	*F:* GGACTGAAGCACCTGTTGTGCA *R:* TCCTGAGTCTCCCAAGGCATTC
*GAPDH*	*F:* GTCTCCTCTGACTTCAACAGCG *R:* ACCACCCTGTTGCTGTAGCCAA

## Data Availability

Not applicable.
